# Mr. Rootsey's Letter to A. T. Thomson, Esq.

**Published:** 1816-04

**Authors:** Samuel Rootsey

**Affiliations:** Bristol


### *To the Editors of the London Medical and Physical Journal.*

GENTLEMEN,

A REVIEW of my Dispensatory having appeared in the last number of the Medical Repository, in which the writer has spoken of my book as "above his comprehension," I am anxious to obtain the favour of your allowing me the use of your pages for my vindication, as the question at issue is one in which I am inclined to think the medical world feel deeply interested. It is no other than this, What shall be the future language of pharmacy?—I should, therefore, thank you to insert the subjoined letter to Mr. Thomson, and am, sir, with great respect, your much obliged and obedient servant, SAMUEL ROOTSEY.

*Bristol; March* 5, 1816.

